# What Makes a Floor Slippery? A Brief Experimental Study of Ceramic Tiles Slip Resistance Depending on Their Properties and Surface Conditions

**DOI:** 10.3390/ma14227064

**Published:** 2021-11-21

**Authors:** Ewa Sudoł, Marcin Małek, Mateusz Jackowski, Marcin Czarnecki, Cezary Strąk

**Affiliations:** 1 Construction Materials Engineering Department, Instytut Techniki Budowlanej, Filtrowa 1, 00-611 Warsaw, Poland; e.sudol@itb.pl (E.S.); m.czarnecki@itb.pl (M.C.); c.strak@itb.pl (C.S.); 2 Faculty of Civil Engineering and Geodesy, Military University of Technology, Gen. Sylwestra Kaliskiego 2, 00-908 Warsaw, Poland; mateusz.jackowski@wat.edu.pl

**Keywords:** slip resistance, ceramic tiles, ramp test, acceptance angle, sliding friction coefficient, surface properties, surface conditions

## Abstract

The safety of the use of construction facilities should be a priority in today’s busy world, where it is not difficult to get involved in an accident. Most of them, due to the pace at which we live today, are caused by slips, trips, and falls. This work presents a detailed analysis of the resistance of ceramic floors to these events, taking into account the surface properties and conditions (dry/wet), which, as presented, have a significant impact on the final slip resistance values. This study also investigates the relationship between surface roughness and anti-slip properties. According to the obtained results, it can be concluded that the surface roughness is not the main determinant of slip resistance, and the final value of it is influenced by many components that should be considered together and not be neglected when designing the surface finish. Furthermore, based on experimental measurements, it can be noted that the highest slip resistance in both wet and dry conditions showed the unglazed tiles with lapatto finish and the glazed tiles without any extra finish.

## 1. Introduction

With normal maintenance, buildings must meet basic requirements not only in terms of fire protection, load-bearing capacity, and stability but also in terms of operational safety. Obviously, construction objects must be designed and constructed in such a way that the loads that may affect them during their construction process and use do not lead to the collapse of the entire object or its part and do not cause its significant deformation or damage disproportionate to its cause [1,2]. Therefore, for the sake of human safety, construction works should also be designed and constructed in such a way that they do not pose an unacceptable risk of accidents or damage in use or operation, such as slips, trips or falls, which may take place during use [3,4,5]. For this purpose, the materials used, especially on communication routes in buildings, should not only be non-flammable and not spread fire but additionally, these routes should be made of materials that do not pose a risk of slipping, tripping, or falling [6].

One of the most commonly used material groups nowadays is ceramic, next to other engineering materials such as polymers, metals, composites, and alloys [7,8,9]. It has great resistance to high temperatures and chemical agents, good dielectricity and insulation, high hardness, and fire resistance. Ceramic also has high strength [10,11], which in some cases is equal to steel or natural stones. However, due to its low tensile and bending strength, it is used as a material that works mostly under compression. Additionally, ceramics is a material susceptible to impact due to its high brittleness and low resistance to mechanical and thermal shocks. Thanks to its properties, this material has been used in metallurgy [12], the energy sector [13], space industry [14,15], nuclear energy sector [16], and construction [17,18]. Ceramic products are very often used in places exposed to weather conditions, aggressive substances, or particularly high temperatures. Therefore, this material can be safely used inside buildings. Due to this, ceramic refractory materials in the form of energy-saving covers, tapes, ceramic mats, insulating bricks, or ceramic plates are more and more often made.

The current use of ceramics inside buildings, next to the construction of brick walls, is the floor finishing layer. This, however, has to provide the right properties to prevent slips, trips, or falls which can cause injuries to the human body and to present the best performance of building [19]. Between 2019 and 2020 alone, there were about 200,970 reported cases of a non-fatal injury caused by slips, trips, or falls (Labour Force Survey, Newport, UK) that could have been prevented by the appropriate choice of surfaces with the correct slip resistance. This term can be defined as a share of the surface itself in the total surface friction. More vividly, it can be said that it reflects the adhesion of pedestrian footwear to the floor. The factors influencing the surface friction and hence the slip resistance of the surface include the speed at which a pedestrian moves across the floor, the texture and geometry of the floor, the properties of the footwear, changes in the conditions of use, in particular the influence of weather conditions, and cleaning and pollution of the floor [20]. The insufficient slip resistance of the floor carries a risk of slipping, tripping, and falling, the consequences of which can be very serious, and the after-injury treatment itself is very often long and highly expensive.

This problem was investigated in the research of Liu et al. [21] as they tested not only the influence of the finishing layer but also the influence of shoes, dirt, and the angle of inclination of the surface on the friction, and thus the anti-slip properties of the surface. In their work, they tested, inter alia, unglazed ceramic tiles, both flat and with a formed profile. The contamination conditions included dry-, wet-, and glycerol-contaminated conditions at substrate slope angles of 0°, 5°, and 10°. As shown by their results, tiles with a molded profile showed greater anti-slip properties than smooth tiles. The influence of the surface finish on the slip resistance was also investigated by Coşkun and Sarıışık [22]. The authors tested existing surfaces in institutional buildings and public places such as hospitals, schools, shopping centers, and universities, and concluded that even now, part of them does not allow for the safe movement of people, especially in wet conditions, as the ST2, ST9, ST12, ST13, ST14, and ST15 surface samples had less than 0.20 dynamic friction coefficients.

As most public areas in buildings, e.g., staircases, corridors, bathrooms, storage rooms, are covered with ceramic tiles today, it is necessary to present a compact study that would define the safety profile of these tiles depending on their initial parameters. Our research answers this need and presents the complex slip resistance research of ceramic tiles, taking into account the method of external surface finish, surface conditions (dry and wet), roughness, and the test method itself.

## 2. Materials

**Figure 1:**
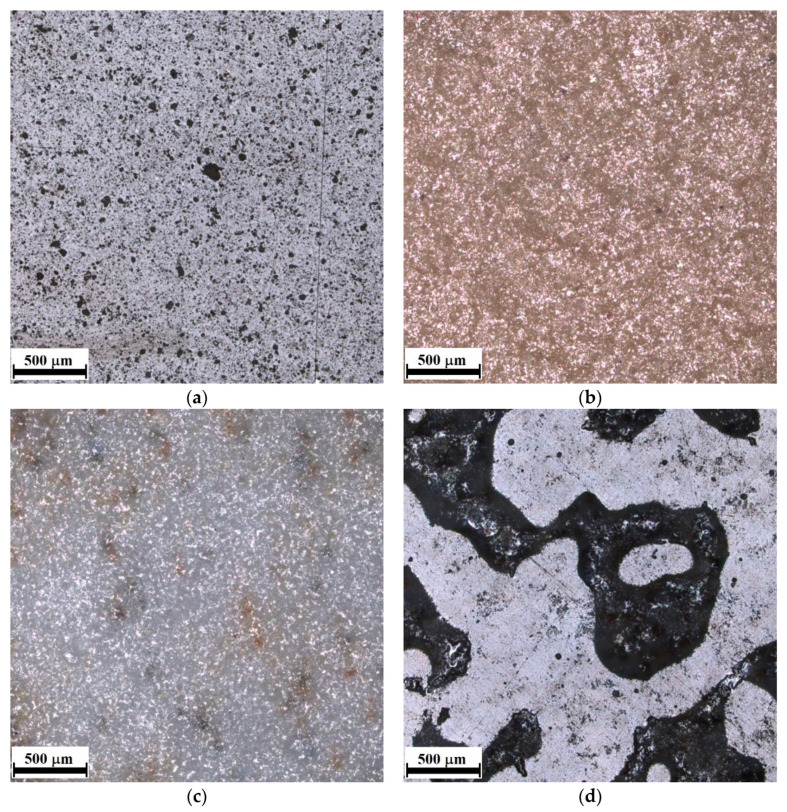

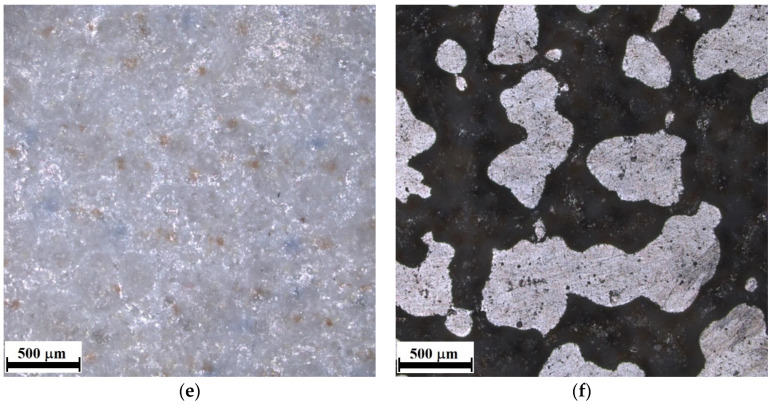
Optical microscopy (OM) photos of ceramic tile outer structure: (**a**) S1, (**b**) S2, (**c**) S3, (**d**) S4, (**e**) S5, and (**f**) S6 sample.

In this research, dry-pressed ceramic tiles, according to EN 14411:2016 [23], were used for the tests. All tested materials had low water absorption E_b_ ≤ 0.5% (group BI_a_), and either a glazed (GL) or unglazed (UGL) surface. The tiles were intended for indoor and outdoor use, including flooring, and had one of four surface finishes: pre-levigato (PLG), levigato (LG), lapatto (LP), or none (N). The pre-levigato finish was made by polishing before burning so the surface of the tiles was smoothed before being placed in the furnace. In contrast, the levigato and the lapatto finish surfaces were polished after burning. In the case of lapatto (semi-polished) tiles, however, the polishing process was interrupted at the moment when the most convex elements of the tile acquired a gloss, while the deeper layers of the structure did not come into contact with the polisher. The plaques were all approximately 600 mm × 600 mm in size and had a thickness ranging from 9 to 10 mm. The characteristics of the tiles, taking into account the surface treatment technology and the roughness determined in the study (LEXT OLS4000, Olympus, Tokyo, Japan), expressed as the arithmetic means of the profile deviation from the mean line (R_a_) and the roughness height according to ten profile points (R_z_), are presented in Table 1. In total, six different types of ceramic tiles have been tested, and their outer structures (LEXT OLS4000, Olympus, Tokyo, Japan) are presented in Figure 1. Before tests, all tiles were cleaned from any dust.

## 3. Methods

### 3.1. The Pendulum Test

**Figure 2:**
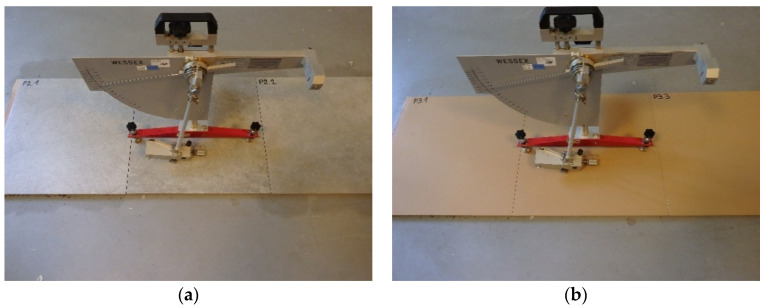
The pendulum test on samples: (**a**) S4 in wet conditions, (**b**) S2 in dry conditions.

The pendulum test was carried out in accordance with CEN/TS 16165:2012 [24] Annex C. In this study, a British pendulum with a CEN slider-type 57 made of 55–61 international rubber hardness degrees (IRHD) rubber 76.2 mm wide, and a 126 mm sliding length was used (WESSEX, Aldershot, UK). The device was calibrated prior to testing with glass, polishing paper, and a reference plate. Conducted research consisted of determining the energy loss of the slider due to friction of the tested surface. The friction force between the slider and the tested surface was determined by measuring the deflection of the pendulum during the slider’s movement, using a calibrated scale (C scale). The slip resistance thus determined was denoted as pendulum test value (PTV). Furthermore, the test was performed on each type of ceramic tile in dry conditions, i.e., after seasoning the samples in laboratory conditions (21 °C temperature and 40% air humidity) and in wet conditions, i.e., after abundantly wetting both the sample and the slider with a sodium lauryl sulfate solution, as seen in Figure 2. Three samples of each type were tested, with 10 measurements in dry conditions and 10 measurements in wet conditions. In total, 360 measurements were made.

### 3.2. The Acceptable Angle Test

**Figure 3:**
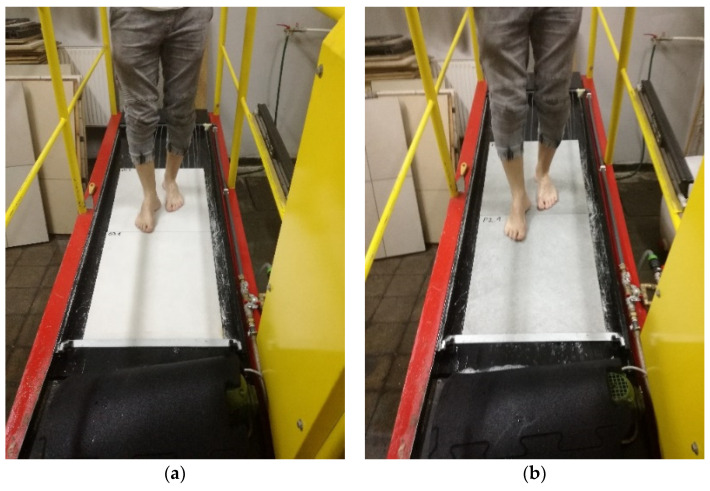
The acceptable angle (ramp) barefoot test on (**a**) S5 and (**b**) S4 samples in wet conditions.

The acceptable angle test, also known as the ramp test, was performed in accordance with CEN/TS 16165:2012 [24] Annex A, barefoot method. During the test, the maximum angle of inclination of the sample in relation to the level (α_b_) at which a person walking on the ramp (Gabrielli SRL, Florence, Italy) with wet feet on the floor covered with the sodium lauryl sulfite solution felt confident was determined (Figure 3a,b). The person walked on the sample straight forward and backward. During the test, the angle of inclination of the sample changed from the horizontal position to the inclination at which the person performing the tests ceased to feel confident and could not continue to walk. The measurements were carried out by two independent researchers. The subjectivity of their experiences was reduced using calibration coverings, and the resulting corrections were incorporated into the acceptance angle value. Three standard coverings were used for the calibration process. The coverings’ acceptance angles were 11.5, 18.5, and 23.9°, respectively. Each person walked on each standard covering three times, and the mean calibration acceptance angle values were determined. Each individual correction value Δα was calculated as a difference between the coverings’ acceptance angle and the calibration acceptance angles. Each individual correction value Δα was less than the critical differences (≤2.1°). If one of the absolute values was greater, the test person in question would be excluded from the test. Correction value (Dj) was calculated from the values obtained from the calibration coverings’ values. The calculation of Dj was carried out as follows:

(1) $$D_{j}=\Delta \alpha -\frac{1}{\sqrt{2}}$$

The correction value was taken into account in the calculation of the acceptable angle, thus eliminating the subjective influences on the test. Each researcher made 10 measurements on each sample. A total of 120 measurements were made, and based on the value of the determined angle α_b_, each material was classified in accordance with PN-EN 13451-1:2020 [25].

### 3.3. The Sliding Friction Coefficient Test

**Figure 4:**
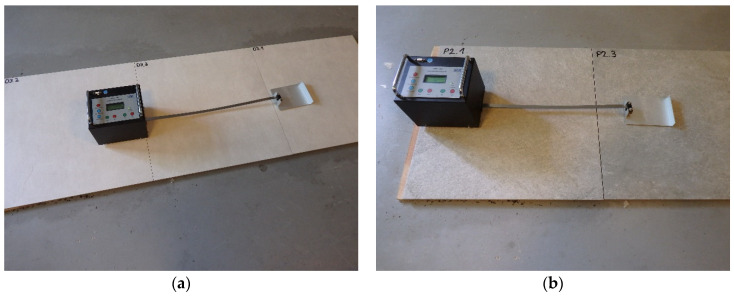
The sliding friction coefficient test on (**a**) S5 and (**b**) S4 samples in dry conditions.

The dynamic coefficient of friction (μ) was tested in accordance with CEN/TS 16165:2012 [24] Annex D on each type of ceramic tile, using a tribometer (GTE Industrieelektronik GmbH, Viersen, Germany) (Figure 4). The device was equipped with sliders imitating shoe heels, exercising a total contact pressure of 9 ± 1 N/cm^2^ when in the static state. A set of three SBR rubber sliders with a density of 1.23 g/cm^3^ and a hardness of 50 on the Shore-D scale was used for the first measurement series, and a set of three leather sliders was used for the second measurement series. Before testing, the device was calibrated with reference substrates (glass, HPL plate, and ceramic tile). The device, while moving on the tested surface, registered the friction force between the slider and the sample. On this basis, the dynamic coefficient of friction (μ), which is the quotient of the friction force and the pressure of the presser foot on the surface, was calculated. The device recorded the data collected while traversing two intersecting paths, each with a length of 1 m. Each type of sample was tested 10 times in dry conditions and 10 times in wet conditions. A total of 120 measurements were made.

## 4. Results

### 4.1. The Pendulum Test

**Figure 5:**
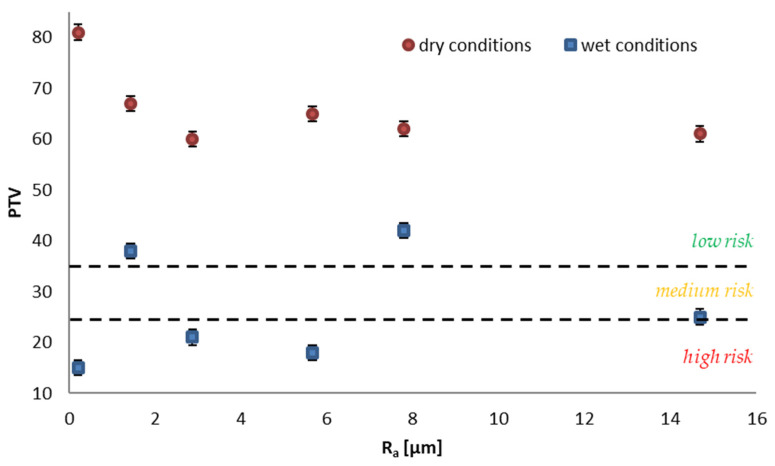
Slip risk classification according to Health and Safety Executive [27] depending on the roughness of surface in wet and dry conditions.

The average values of the slip resistance of ceramic tiles are shown in Table 2. Analyzing the obtained pendulum test values (PTV) in terms of The UK Slip Resistance Group [26] criteria, it can be concluded that all tested solutions in the dry state were characterized by PTV ≥ 36, which allows them to be considered as having a low risk of slippage (Figure 5). No influence of the surface finish or roughness on the dry slip resistance class according to the PTV value was observed. When the condition of the floor changes from dry to wet, a clear reduction in PTV can be found. Only for the S2 series tiles–unglazed, semi-polished (lapatto), and S5–glazed unpolished, a PTV level greater than 36 was maintained (Figure 5). This means that when wet, the risk of slipping increases to medium for the S6 series–unglazed semi-polished (lapatto) tiles with a Ra of 14.7 μm and to high risk in the case of other solutions [27]. Particularly noteworthy are the unglazed polished tiles, both pre-levigato (S1 series) and levigato (S3 series) and semi-polished tiles with Ra 5.7 μm (S4 series), where wetting with water decreased the PTV value from 81 to 15 units, from 60 to 21 units, and from 65 to 18 units, respectively, which may lead to the conclusion that the scope of application of these tiles should be limited to zones where they are not intended for use in wet conditions, i.e., with the exception of, for example, sanitary facilities or entrance zones to buildings.

It is noticeable that the roughness of the surface did not play such an important role in shaping the PTV value as the technology of its finish, as seen in Figure 5. The distribution of values is rather random in the case of wet conditions, and in the case of dry conditions, it fluctuates at similar values of 60 to 67 (except for sample S1, where the value is much higher–81 PTV). Similar conclusions were reached by Terjek et al. [28], who conducted research on the slip resistance for 14 solutions of unglazed ceramic tiles and 14 solutions of glazed tiles. The author pointed out that the tested solutions are characterized by very different slip resistance. In dry conditions, the PTV values were from 55 to 92 for unglazed tiles and from 58 to 91 for glazed ones, and in wet conditions from 15 to 60 and 16 to 64, respectively. Additionally, a similar drop in PTV value in wet conditions was reported by Strąk et al. [29]. They tested three types of sport surfaces made from recycled rubber and noted about a 50% drop in the slip resistance when wet. Furthermore, Sudoł et al. [30] conducted research on granite tiles and came to the same conclusions. The highest decrease in values they obtained was for polished granite tiles (22 PTV in wet conditions), and the final classification showed a “high-risk” class. As presented, wet conditions significantly lower slip resistance of the surface, and this is not connected to the material that it is made of but rather its surface finish.

### 4.2. The Acceptable Angle Test

**Figure 6:**
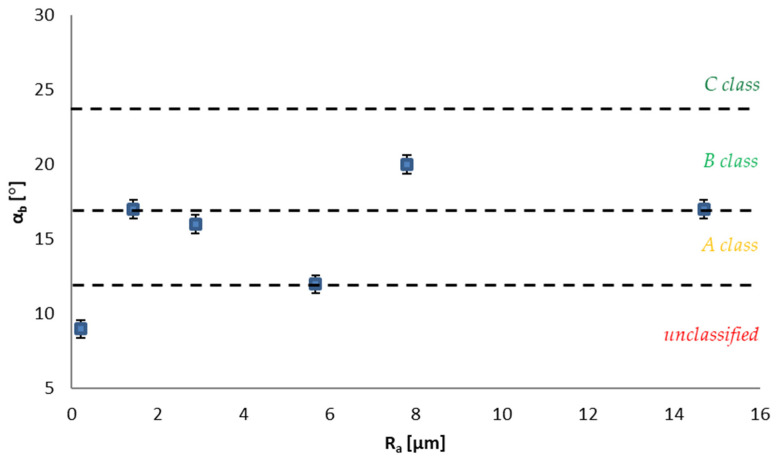
Slip resistance classification according to EN 13451-1:2020 [25] depending on the roughness of surface.

The unfavorable effect of water on the anti-slip properties of the tested tiles was also clearly visible in the examination of the acceptable angle by using the barefoot method (Figure 6). The obtained value of α_b_ for the S1–unglazed tiles polished in the pre-levigato technology–at the level of 9° did not allow their classification on a scale appropriate for floor tiles [25]. The values set for the S4 series–unglazed semi-polished tiles (12°), S3–unglazed polished tiles in the levigato technology (16°) and S6–glazed semi-polished tiles, and S5–unpolished glazed tiles (17°) classify them as the lowest class A (Figure 6). As a consequence, the listed tiles should not be used in buildings where the floor is wet and people walk without shoes on, e.g., swimming pool facilities. Class A tiles may be installed only in swimming pool basins with a water depth of more than 800 mm, if the floor has no slope [25]. As presented in Figure 6, only the S5 series tiles can be considered as class B (20° acceptable angle), which extends the scope of their use in swimming pool facilities to the pool basins with a water depth of 0 mm up to 1350 mm with the 8° or less floor slope and surfaces surrounding the pool basin that are exposed to water during use [21]. None of the tested solutions obtained an acceptable angle of more than 24°, which would allow them to be classified in the highest class C, and thus they can be considered useful for floors with an inclination greater than 8° in basins with a water depth from 0 mm to 1350 mm and facing stairs, starting platforms, etc., in swimming pool facilities [25]. There was an increase in the value of the acceptable angle with the increase in the R_a_ value, but this was not applicable to the S6 series. In the already mentioned studies by Terjek et al. [28] in the study of the angle acceptable for bare feet, they obtained even lower values, ranging from 7° to 12° for unglazed tiles and from 6° to 11° for glazed tiles, questioning the suitability of the considered solutions for use in barefoot-used floors in the presence of water.

Comparing the acceptable angle test results with the results of PTV in the wet state, no clear relationship was observed. Solutions for which the highest PTV values in the wet state were obtained at a level above 36 units; the S2 and S5 series showed the highest values of the acceptable angle with α_b_ 17° and 20°, respectively, but at a level very similar to the one also established for the S3–16° or S6–17° series, which, in the PTV test in the wet condition, was characterized by a slip resistance appropriate for a high and medium slip risk, respectively, when footwear is worn [26]. It can be noted that there is a very large discrepancy in the results determining the safety of movement on the tested surfaces. Therefore, when materials are used on surfaces where people can walk barefoot, conducting tests using more than one method is recommended. Furthermore, the final classification should be based on the lowest result, or there should be appropriate restrictions introduced on the applicability of the tested material, e.g., excluding bathing and swimming pools.

Analyzing Figure 6, an analogy to the results obtained for the pendulum test can be found. In this case, the values were distributed randomly as well, which can be proven by the same parameters obtained for samples S2 and S6 with a roughness of 1.43 and 14.69 μm, respectively, which presents a similar trend to the values obtained for granite tiles in the study by Sudoł et al. [30]. Their results of the acceptable angle also had a random distribution and did not depend on the surface roughness. Furthermore, it can be said again that the surface roughness does not play such an important role in shaping the results as the technology of its finishing layer.

### 4.3. The Sliding Friction Coefficient Test

**Figure 7:**
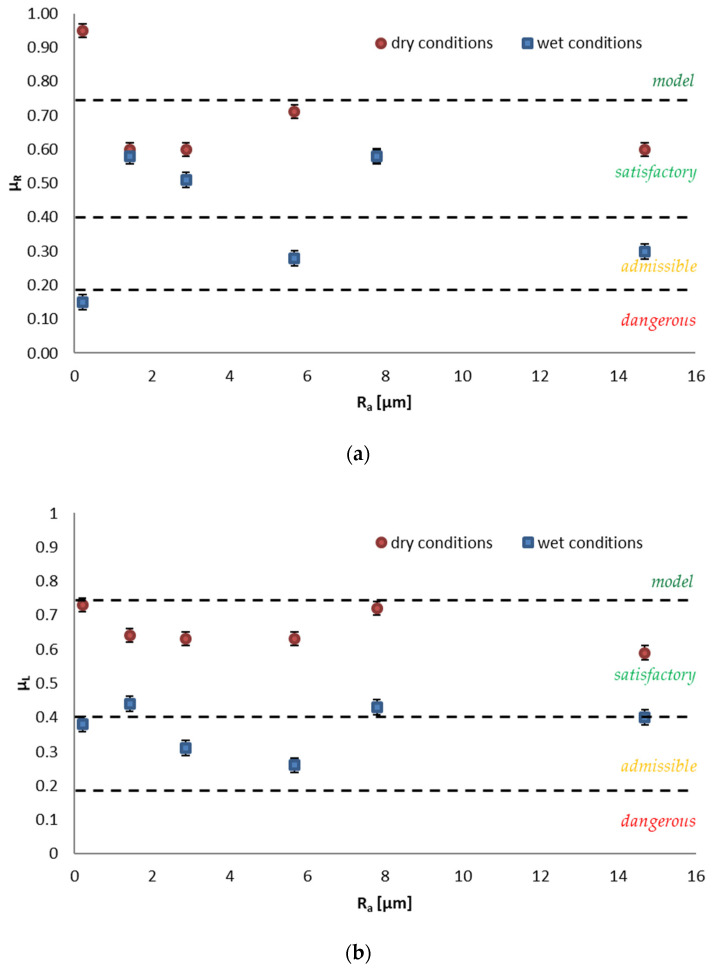
The sliding friction coefficient test results classification according to Nemire et al. [3] and Bellopede et al. [31] depending on the roughness of surface for (**a**) rubber sliders and (**b**) leather sliders in wet and dry conditions.

The results of the dynamic coefficient of friction obtained in the test with the rubber sliders set (μ_R_) and in the test with leather sliders set (μ_L_) are shown in Figure 7a,b, respectively. The trend in the μ_R_ and μ_L_ values is similar to that observed for the PTV tests. The highest values of μ_R_ in a dry state were recorded for the S1 series—0.95, which allows for classification at the reference level. Comparable μ_R_ results were obtained in the dry state for the remaining series: 0.58—S5, 0.60—S2, S3 and S6, and 0.71—S4, which indicates a satisfactory slip resistance that is in accordance with the criteria [3,30]. After the transition from dry to wet, only plates of the S2 series with a μ_R_ value of 0.58, the S3 series with a μ_R_ value of 0.51, and S5 with a μ_R_ value of 0.58 were maintained in the above class. The S4 series with a μ_R_ value of 0.28 and the S6 series with a μ_R_ value of 0.30 have a slip resistance at the acceptable level, while the S1 series with a μ_R_ value of 0.15 should be considered dangerous. The results of the dynamic coefficient of friction obtained in dry conditions with the μ_L_ leather sliders set correlate with μ_R_. The results of μ_L_ obtained in wet conditions are slightly different. In the case of the S1 and S6 series, an increase in the value of the dynamic coefficient of friction was noted at the level of 0.38 and 0.40, which allows for a higher classification of the solutions as admissible and satisfactory, respectively. In relation to the S3 series, the values of μ_L_ were lower than μ_R_, indicating a lower class of slip resistance.

In the case of the friction coefficient for rubber sliders, the distribution of values was random. No relationship was observed between the roughness and the friction coefficient. However, for leather sliders, the obtained values oscillated within constant limits, i.e., between 0.73–0.59 for dry conditions and 0.26–0.44 for wet conditions.

## 5. Discussion

In general, a surface that is clean and dry poses very little slip risk. When we add dust or other dry contaminants, such as drywall or concrete dust, these tiny particles can act like ball bearings, when they are round, and create a high slippery situation [21,32,33]. Nowadays, most floors are polished, glazed, or even waxed [34] to make them very smooth, glossy, and easy to clean. However, what happens when these kinds of floors get wet? As presented in this study, even the average rubber shoes can move safely on a smooth, dry, and polished surface, but this changes when the floor gets wet. The highest drop in safety was reported for S1 samples that were unglazed and polished before firing (pre-levigato finish). The values of the sliding friction coefficient decreased from 0.95 to 0.15 for them. This disproportion was lower when leather sliders were used, as the values went down from 0.73 to 0.38. This trend was also noted during the pendulum test. Due to that, it can be concluded that unglazed PLG finish tiles (S1) are the most dangerous in wet conditions from all tested tiles; however, they also achieved the highest results in all tests in dry conditions. On the other hand, S2 and S5 samples showed the lowest difference in obtained values in wet and dry conditions. This was especially noticeable during the sliding friction coefficient test, as the values for rubber slider were equal for the S5 sample (0.58) and about equal for the S2 sample (0.60 and 0.58) in dry and wet conditions, respectively. A slightly bigger disproportion was observed when testing with leather sliders, but this trend was maintained and also reported after the pendulum test. Both samples kept their low risk classification. As a result, it can be concluded that unglazed tiles with lapatto (LP) and glazed tiles with no (N) finish have the best potential to keep their anti-slip properties in both wet and dry conditions. Contrastingly, a drop in classification, was reported for unglazed tiles with lapatto (LP) finish (samples S4 and S6). The polished layer made after the firing process caused a decrease in anti-slip resistance in both cases, as both the values and the final slip resistance classification went down. It changed from “low risk” to “medium risk” (S6) or even “high risk” (S4) in the pendulum test and from “satisfactory” to “admissible” (S4 and S6) in the sliding friction coefficient test. Additionally, for sample S3, inconclusive results were obtained, because in the case of rubber sliders, the results in dry and wet conditions were similar, while for the leather sliders, the results differed by 0.32. This difference caused the classification to fall to “admissible” due to the need to use the lowest value as a benchmark. In the case of the acceptable angle test and the pendulum test, one of the lowest wet classes were also obtained (“A class” and “high risk”, respectively) what proves the low slip resistance of these tiles in wet conditions. The same conclusion was reached by Chen et al. [35] as they tested six different types of floors and six different types of liquids and their effect on the slip resistance of the surface. They pointed out that when the floor is wet, the higher the viscosity of the liquid, the longer the time to connect the shoe with the floor, and the higher the risk of slipping and falling down. Chen et al. [35] reported that the best strategy to prevent injuries should be keeping the floor dry all the time, which in some places is unfortunately impossible. That is why it is necessary to get a better look at the external structure of the floor to find the slip indicators.

**Figure 8:**
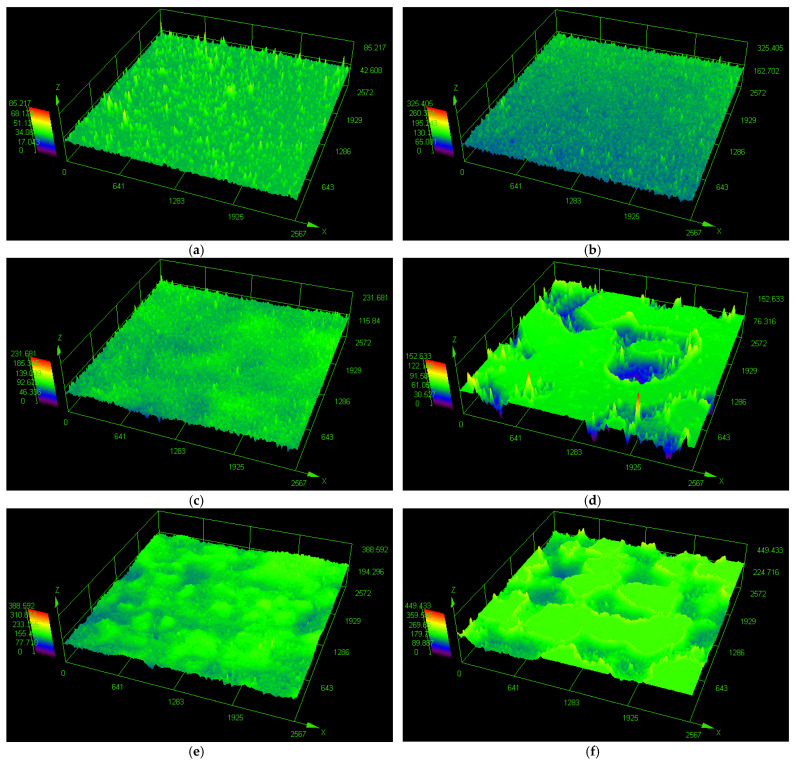
Optical maps of the tile surface: (**a**) S1, (**b**) S2, (**c**) S3, (**d**) S4, (**e**) S5, and (**f**) S6.

The influence of the external structure of the tiles is visible in the tests performed. The surface finish has an influence, above all, on the friction exerted between the shoe and the tile and its possible reduction or disappearance as a result of surface wetting. In order to thoroughly analyze the reasons for the differences between the results in the dry and wet state, the optical profilometer LEXT OLS4000 (Olympus, Tokyo, Japan) was used, and pictures of the external structure of the finishing layers of individual tiles were taken, as seen in Figure 8.

When a floor is smooth and wet, we run into the risk of hydroplaning on top of the water, which is extremely difficult to recover from. If a person hydroplanes for more than a couple of inches, there is a great chance of falling down and getting injured. This changes when the tile has sharp points on the walking surface. This way, there is greater traction between the shoe and tile, even in wet conditions, but the points must be sharp and not rounded off bumps. They, on the other hand, do not help a person to move on a wet floor at all and can be very slippery (Figure 8a). Thousands of slip accidents have been reported on these types of rounded bumpy surfaces [3]. Mentioned sharp points can only be seen at a microscopic level making it hard to tell with the naked eye which floors will be safe and which will be slippery. As presented in Figure 8b, small sharp points distributed evenly on the whole surface contribute to greater traction even in wet conditions, which was reported during slip resistance tests in this study (S2). They, however, must be about the same high (around 130 μm) and distributed evenly; otherwise, they do not maintain the anti-slip parameters of the floor in wet conditions, which was reported for S3 sample, as seen in Figure 8c. The only other way we could obtain traction in wet conditions was through tiny pores edged into the surface (Figure 8e). The pores filled with water acted like suction cups and gave us a grip on wet floors, when sufficiently deep (around 60 μm). This phenomenon was observed for S5 samples, as they maintained their properties in dry and wet conditions. However, not all pores work like this. The bigger the pores, the lower the ability to keep the anti-slip properties when wet, as seen in Figure 8d,f. In this case, greater water areas cause a hydroplaning effect, and a person is prone to loss of balance and collapse, which was reported for S4 and S6 samples. So, when the floor is expected to get wet, we need sharp points on top of the surface or tiny pores within the surface to give us good traction. This shows that in this study, a clear correlation between surface finish and slip resistance has been found.

## 6. Conclusions

The slip resistance of a floor should be tested regularly to ensure that the floor safety has not changed through poor maintenance practices or through general wear. This study analyzed the slip resistance of six different types of ceramic tiles to clearly present slip resistance indicators of ceramic tiles. Due to our own experimental research, the following conclusions can be made:

1. Slip resistance is highly connected to surface conditions, as the values of the dry surfaces were about 1.5–5.4 times higher than those of the wet surfaces for the pendulum tests. This was also confirmed by the acceptable angle barefoot test and the sliding friction coefficient test.
2. There is a clear correlation between the surface finish and slip resistance of ceramic tiles.
3. The unglazed tiles with lapatto surface (S2) and glazed tiles with no finish (S5) have the best potential of keeping their anti-slip properties in both dry and wet conditions, as they showed 67/38 PTV and 62/42 PTV, respectively, in the pendulum test.
4. The unglazed tiles with pre-levigato finish (S1) have the lowest slip resistance in wet conditions: 15 PTV for the pendulum test, 9° for the acceptable angle test and sliding friction coefficients of 0.15 and 0.38 for rubber and leather sliders, respectively.
5. No clear correlation was observed between the surface roughness and its slip resistance, because the obtained results (in all applied test methods) had a random distribution.
6. Ceramic tiles should be tested with more than one method due to the difference in values between methods. Therefore, the final safety classification should be made on the basis of the lowest slip resistance values obtained.
7. The use of tested tiles, for which the classes A and B were established, in wet areas used with bare feet should be significantly limited.

## Figures and Tables

**Table 1:** The properties of tested ceramic tiles.

Sample Symbol	Surface Type	Surface Finish	R_a_ [µm]	R_z_ [µm]
S1	UGL	PLG	0.21	4.26
S2	UGL	LP	1.43	10.41
S3	UGL	LG	2.87	15.33
S4	UGL	LP	5.67	30.50
S5	GL	N	7.79	42.99
S6	UGL	LP	14.69	64.82

**Table 2:** The slip resistance tests results.

Sample Symbol	The Pendulum Test Value (PTV) [-]		The Acceptable Angle (α) [°]	The Sliding Friction Coefficient (µ) [-]			
S1	81 ± 1 ^1^	15 ± 1 ^2^	9 ± 1	0.95 ± 0.02 ^1,3^	0.15 ± 0.01 ^2,3^	0.73 ± 0.02 ^1,4^	0.38 ± 0.01 ^2,4^
S2	67 ± 1 ^1^	38 ± 1 ^2^	17 ± 1	0.60 ± 0.01 ^1,3^	0.58 ± 0.01 ^2,3^	0.64 ± 0.01 ^1,4^	0.44 ± 0.01 ^2,4^
S3	60 ± 1 ^1^	21 ± 1 ^2^	16 ± 1	0.60 ± 0.01 ^1,3^	0.51 ± 0.01 ^2,3^	0.63 ± 0.01 ^1,4^	0.31 ± 0.01 ^2,4^
S4	65 ± 1 ^1^	18 ± 1 ^2^	12 ± 1	0.71 ± 0.02 ^1,3^	0.28 ± 0.01 ^2,3^	0.63 ± 0.01 ^1,4^	0.26 ± 0.01 ^2,4^
S5	62 ± 1 ^1^	42 ± 1 ^2^	20 ± 1	0.58 ± 0.01 ^1,3^	0.58 ± 0.01 ^2,3^	0.72 ± 0.02 ^1,4^	0.43 ± 0.01 ^2,4^
S6	61 ± 1 ^1^	25 ± 1 ^2^	17 ± 1	0.60 ± 0.01 ^1,3^	0.30 ± 0.01 ^2,3^	0.59 ± 0.01 ^1,4^	0.40 ± 0.01 ^2,4^

^1^ dry conditions, ^2^ wet conditions, ^3^ rubber sliders, ^4^ leather sliders.

## Data Availability

Data are contained within the article.
